# Foliar phloem infrastructure in support of photosynthesis

**DOI:** 10.3389/fpls.2013.00194

**Published:** 2013-06-14

**Authors:** William W. Adams, Christopher M. Cohu, Onno Muller, Barbara Demmig-Adams

**Affiliations:** Department of Ecology and Evolutionary Biology, University of ColoradoBoulder, CO, USA

**Keywords:** apoplastic loaders, companion cells, intermediary cells, minor loading veins, phloem loading, photosynthetic capacity, sieve elements, symplastic loaders

## Abstract

Acclimatory adjustments of foliar minor loading veins in response to growth at different temperatures and light intensities are evaluated. These adjustments are related to their role in providing infrastructure for the export of photosynthetic products as a prerequisite for full acclimation of photosynthesis to the respective environmental conditions. Among winter-active apoplastic loaders, higher photosynthesis rates were associated with greater numbers of sieve elements per minor vein as well as an increased apparent total membrane area of cells involved in phloem loading (greater numbers of cells and/or greater cell wall invaginations). Among summer-active apoplastic loaders, higher photosynthesis rates were associated with increased vein density and, possibly, a greater number of sieve elements and companion cells per minor vein. Among symplastic loaders, minor loading vein architecture (number per vein and arrangement of cells) was apparently constrained, but higher photosynthesis rates were associated with higher foliar vein densities and larger intermediary cells (presumably providing a greater volume for enzymes involved in active raffinose sugar synthesis). Winter-active apoplastic loaders thus apparently place emphasis on adjustments of cell membrane area (presumably available for transport proteins active in loading of minor veins), while symplastic loaders apparently place emphasis on increasing the volume of cells in which their active loading step takes place. Presumably to accommodate a greater flux of photosynthate through the foliar veins, winter-active apoplastic loaders also have a higher number of sieve elements per minor loading vein, whereas symplastic loaders and summer-active apoplastic loaders have a higher total number of veins per leaf area. These latter adjustments in the vasculature (during leaf development) may also apply to the xylem (via greater numbers of tracheids per vein and/or greater vein density per leaf area) serving to increase water flux to mesophyll tissues in support of high rates of transpiration typically associated with high rates of photosynthesis.

## INTRODUCTION

Foliar vasculature, as the network of tissue linking the leaf’s chloroplast-containing mesophyll cells to the rest of the plant, should be expected to display some concomitant adjustments to those of photosynthesis during leaf development. Correlations between xylem hydraulic conductivity and photosynthesis (e.g., Hubbard et al., 2001; Brodribb et al., 2005; Nardini et al., 2005; Sack and Holbrook, 2006) have thus far led investigations of the relationship between leaf venation and photosynthesis (Boyce et al., 2009; Beerling and Franks, 2010; Brodribb and Field, 2010; Brodribb et al., 2010; McKown et al., 2010; Blonder et al., 2011; Walls, 2011). In addition, we (Adams et al., 2005, Adams et al., 2007; Amiard et al., 2005) and others (Ainsworth and Bush, 2011; Nikinmaa et al., 2013) have suggested that the export of carbohydrates from leaves might also be tightly coupled to photosynthesis rate.

Several principal adjustments of foliar phloem (during leaf development) can be envisioned to affect the capacity for carbohydrate export from the respective leaves. For apoplastic loaders, which rely on membrane-spanning proteins [adenosine triphosphatases (ATPases) to actively pump protons into the cell wall space and, e.g., sucrose-H^+^ co-transporters to move sucrose from the apoplast into the phloem], a greater total membrane area of cells engaged in active loading of sugars or sugar alcohols could be achieved via greater cell wall invagination, greater cell size, or greater numbers of cells involved in loading. For symplastic loaders, which rely on diffusion of sucrose into intermediary cells (IC) and active synthesis of larger, raffinose sugars in ICs, ICs of larger sizes may allow (i) a greater volume available for raffinose-synthesizing enzymes and (ii) greater cell wall length available for placement of plasmodesmatal channels for sugar movement. For all leaves, regardless of phloem loading mechanism, a greater flux of sugar-laden phloem sap should be afforded by a greater number of foliar veins per leaf area, larger sieve elements, and/or more sieve elements per vein. How might any of this be related to photosynthesis? Manipulative experiments have clearly shown that photosynthesis can be downregulated in response to inhibition of sugar export from leaves/needles (Krapp et al., 1993; Layne and Flore, 1993; Krapp and Stitt, 1995; Myers et al., 1999; Paul and Foyer, 2001; Urban and Alphonsout, 2007). This downregulation occurs via sugar repression of photosynthetic genes and lowers intrinsic (light- and CO_2_-saturated) photosynthetic capacity for photosynthesis – the latter of which should therefore be expected to be directly dependent on the capacity to export the products of photosynthesis from the leaf. In the following, we review evidence indeed indicating such a linkage between the phloem structure of foliar minor loading veins and intrinsic photosynthetic capacity in those leaves.

## LIMITS TO ACCLIMATORY RESPONSES OF FULLY DEVELOPED LEAVES

**FIGURE 1:**
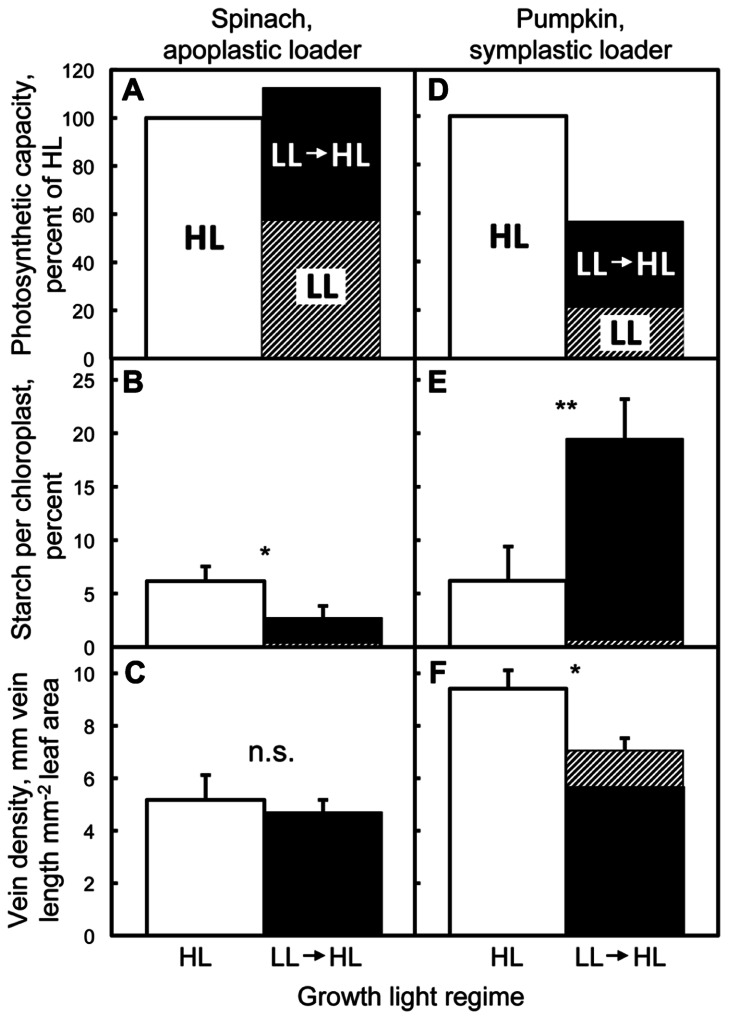
**Photosynthetic capacities, nocturnal retention of starch, and vein densities of fully expanded leaves acclimated to high light (HL), low light (LL), and 1 week after transfer from low to high light.** Photosynthetic capacity (light- and CO_2_-saturated rates of oxygen evolution determined in a leaf disc oxygen electrode at 25°C; Delieu and Walker, 1981) relative to that determined from fully expanded leaves of spinach **(A)** and pumpkin **(D)** grown under HL, fractional area of the chloroplasts filled with starch grains in spinach **(B)** and pumpkin **(E)** quantified from cross-sections of palisade cells (examined with electron microscopy) from fully expanded leaves collected predawn, and vein densities of fully expanded leaves of spinach **(C)** and pumpkin **(F)**. Plants were germinated and grown under a 9-h photoperiod of 150 (spinach) or 100 (pumpkin) μmol photons m^-2^ s^-1^(low light = LL), or under a 14-h photoperiod of 1000 μmol photons m^-2^ s^-1^ (high light = HL), or developed under LL and subsequently transferred for 1 week to HL (LL→HL), all at 25°C/20°C day/night temperature. Only leaves that had expanded fully under LL (hatched portion of the LL→HL column) were characterized 1 week after transfer to HL (filled portions of the LL→HL column). Means (*n* = 3) ± standard deviation depicted in B, C, E, and F. Asterisk (*) and ** indicate significant differences at *p* < 0.05 and *p* < 0.01, respectively, and *n.s*. = not significantly different (Students *t*-test). Data redrawn or recalculated from Amiard et al. (2005). Photosynthetic capacities of leaves from HL plants and LL transferred to HL plants were both significantly higher than leaves of LL spinach plants **(A)**, and photosynthetic capacities of pumpkin leaves were significantly different from each other for all three growth conditions **(D)**.

Amiard et al. (2005) characterized fully expanded leaves and intrinsic photosynthetic capacity of two apoplastically and two symplastically loading species grown under low light (LL), high light (HL), and transferred from LL to HL for 1 week. Intrinsic photosynthetic capacities of the apoplastic loaders pea (*Pisum sativum* L. cv. Alaska) and spinach (*Spinacia oleracea* L. cv. Giant Nobel; **Figure 1A**) were significantly higher in leaves of plants grown under HL compared to LL, and were found to be equally high in mature leaves of LL-grown plants following 1 week after transfer to HL. This full upregulation of photosynthetic capacity in transferred LL-grown leaves to the level seen in HL-grown leaves was presumably facilitated by an increased level of ATPases and/or sucrose-H^+^ co-transporters in the companion cells of spinach and pea, with pea exhibiting significantly higher levels of companion cell wall ingrowths of HL-grown and LL to HL-transferred leaves compared to LL-grown leaves (Amiard et al., 2005). Putative upregulation of the numbers of transport proteins involved in sucrose export would facilitate increased sugar flux out of the leaves Vaughn et al. (2002), preventing accumulation of non-structural carbohydrates in the leaves (**Figure 1B**), and thereby permitting full upregulation of photosynthetic capacity. Vein density per leaf area was not higher in leaves (of these apoplastic loaders) developed in HL compared to LL, and also did not change (in fully expanded leaves) following exposure of LL plants to HL for seven days (**Figure 1C**). Similar results were reported for the additional apoplastic loaders *Arabidopsis thaliana* (L.) Heynhold Col-0 and *Senecio vulgaris* L., both of which also exhibited greater cell wall ingrowths in transfer cells of HL-acclimated leaves compared to LL-acclimated leaves (Adams et al., 2005, Adams et al., 2007; Amiard et al., 2007).

Two symplastic loaders, pumpkin (*Cucurbita pepo* L. cv. Autumn Gold; **Figure 1D**) and *Verbascum phoeniceum* L. (not shown; see Amiard et al., 2005), also exhibited significantly higher photosynthetic capacities when grown under HL compared to LL, but neither species was capable of upregulating photosynthetic capacity to the same high level exhibited by HL-grown leaves following transfer of LL-grown plants to HL for 1 week. Both species also exhibited significantly lower foliar vein densities in LL compared to HL, and vein density remained at that low level in (fully expanded) leaves transferred from LL to HL (**Figure 1F** for pumpkin; see Amiard et al., 2005 for *V. phoeniceum*). It is likely that fixed physical features [low, unchanging plasmodesmata number (determined from electron micrographs of the walls of intermediary cells; Amiard et al., 2005) and foliar vein density] of the route for sugar export in these symplastic loaders limited the ability of LL-acclimated leaves to export increased levels of sugars synthesized by plants transferred from LL to HL, as indicated by an accumulation of starch in pumpkin (**Figure 1E**), presumably preventing full upregulation of photosynthetic capacity.

Spinach (a winter-active annual and apoplastic loader) and *V. phoeniceum* (a biennial symplastic loader) both exhibited upregulation of photosynthetic capacity in response to growth at low versus moderate temperature (Adams et al., 1995; Dumlao et al., 2012). As during the acclimation to light intensity described above, foliar vein density was significantly greater in *V. phoeniceum* leaves that had developed under low versus moderate temperature (matching the greater photosynthetic capacity at low temperature), but was not significantly different between growth temperatures for spinach (Dumlao et al., 2012). As will be shown in the next section, spinach exhibited a greater number of phloem cells per vein when grown at low temperature, presumably providing for greater ATPase activity and sucrose loading of the phloem to facilitate upregulation of photosynthetic capacity at lower temperature. In contrast to the full or partial upregulation of photosynthesis seen in mature leaves of apoplastic and symplastic loaders upon transfer from LL to HL described above, neither spinach nor *V. phoeniceum* exhibited any significant upregulation of photosynthetic capacity in mature leaves 1 week after short-term transfer from moderate to low temperature growth conditions (Dumlao et al., 2012).

## SIEVE ELEMENT AND COMPANION CELL NUMBER ARE RELATED TO PHOTOSYNTHETIC CAPACITY IN SEVERAL WINTER-ACTIVE APOPLASTIC LOADERS

*Arabidopsis thaliana* (L.) Heynhold Col-0 and *S. oleracea* L. cv. Giant Nobel (spinach) germinate in late summer to early autumn, overwinter as a rosette of leaves, followed by bolting and flowering during the lengthening, warmer days of spring. These winter annuals, both of which are apoplastic loaders (Lohaus et al., 1995; Haritatos et al., 2000), were grown under several combinations of growth light and temperatures, i.e., warm (day/night leaf temperatures of 25°C/20°C) versus cool (14°C/8°C) temperature and moderate (400 μmol photons m^-2^ s^-1^) versus high (1000 μmol photons m^-2^ s^-1^) light (9-h photoperiod), leading to leaves with different minor loading vein architecture and photosynthetic capacities. All seeds were germinated under the 25°C/20°C leaf temperature regime and then either maintained under those conditions or, following cotyledon development, transferred to a 19°C/15°C leaf temperature regime for 7 days followed by transfer to the leaf temperature regime for development under cool temperature conditions. In all cases, only leaves that were initiated and had developed fully under the respective conditions were characterized. Plants were maintained under the respective light conditions from germination through development and characterization. This protocol was followed for *A. thaliana* and spinach, as well as all of the other species described below.

**FIGURE 2:**
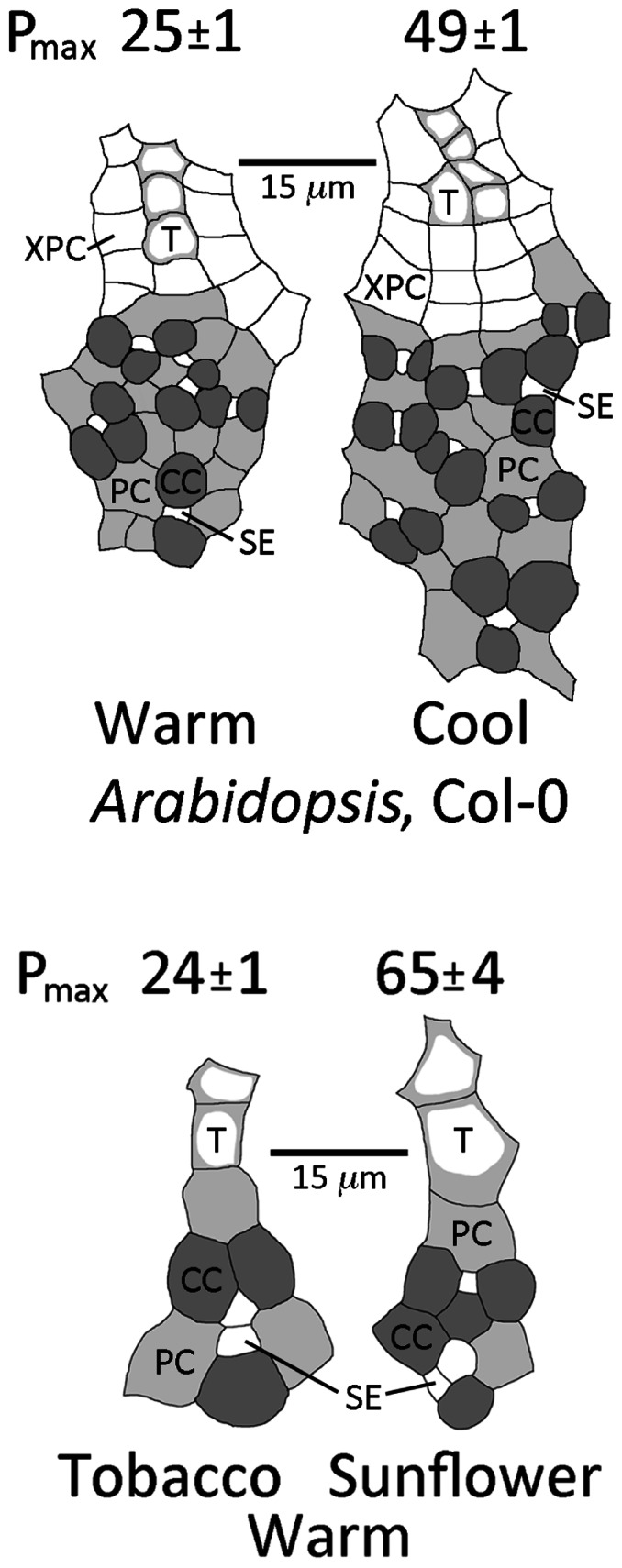
**Sketches of representative minor loading veins from fully expanded leaves of the winter annual *Arabidopsis thaliana* Col-0 grown under a 9-h photoperiod of 400 μmol photons m^–2^ s^–1^ at an average day/night leaf temperature of 25°C/20°C (warm) or 14°C/8°C (cool) and from fully expanded leaves of the summer annuals tobacco and sunflower grown under a 9-h photoperiod of 1000 μ mol photons m^–2^ s^**–1**^ at an average day/night leaf temperature of 25°C/20°C.** Filled dark gray cells are companion cells (CC; 12 and 19 cells in the warm and cool veins of *A. thaliana*, respectively), filled light gray cells are phloem parenchyma cells (PC), small white cells within the gray cells are sieve elements (SE; 6 and 9 cells in the warm and cool veins of *A. thaliana*, respectively), cells with light gray adjacent to the cell walls are tracheids (T; 3 and 5 cells in the warm and cool veins of *A. thaliana*, respectively), and large white cells around the tracheids are xylem parenchyma cells (XPC). Photosynthetic capacities (light- and CO_2_-saturated rates of oxygen evolution, *P*_ max_ in μmol O_2_ m^-2^ s^-1^) ± standard deviation obtained from one leaf from each of four plants (*n* = 4) per growth condition provided above each sketch. Mean photosynthetic capacities between *A. thaliana* leaves grown at the two temperatures, and between tobacco and sunflower leaves grown under the common conditions, were significantly different at *p* < 0.001 (Student’s *t*-test).

For the winter annuals *A. thaliana* and spinach, growth under lower temperature resulted in a greater number of sieve elements, companion cells, and phloem parenchyma cells in the minor loading veins compared to growth at warm temperature, as well as a higher intrinsic capacity of photosynthesis. This difference in phloem cell numbers is illustrated in **Figure 2** for *A. thaliana* grown at two temperatures under moderate light, with a twofold higher photosynthetic capacity (*P*_ max_) in leaves grown at cool versus warm temperature. Growth under HL further accentuated this difference in phloem anatomy; mean (±standard error, *n* = 4 plants) sieve element number per minor loading vein was 11.1 ± 0.3 (ranging between 8 and 14) in leaves grown at 14°C under HL versus 6.8 ± 0.3 (ranging between 4 and 10) in leaves grown at 25° under moderate light (*p* < 0.001, Student’s *t*-test). For spinach, grown under the same conditions as *A. thaliana* above, sieve element number per minor loading vein was 12.0 ± 0.7 in leaves grown at 14°C under HL versus 5.0 ± 0.7 in leaves grown at 25°C under moderate light (*p* < 0.001, Student’s *t-*test). See Cohu et al. (in revision) for an in-depth analysis of the acclimation of foliar vasculature to temperature and light among three ecotypes of *A. thaliana*.

**FIGURE 3:**
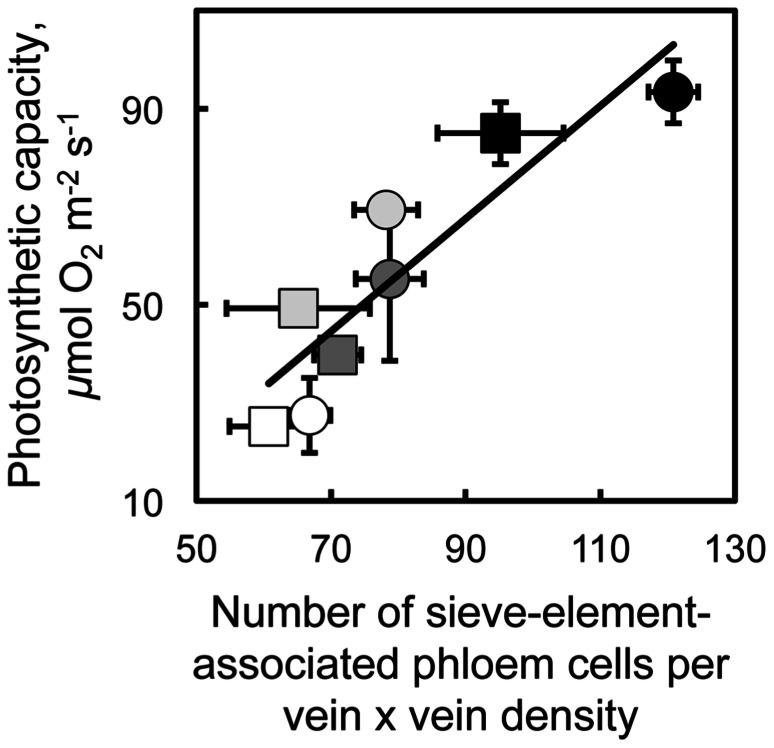
**Relationship between the light- and CO_2_-saturated rate of oxygen evolution (photosynthetic capacity) and the product of vein density times the number of companion and phloem parenchyma cells adjacent to sieve elements in the minor loading veins from fully expanded leaves of the apoplastic loaders *Arabidopsis thaliana* Col-0 (squares) and spinach (circles).**Plants were grown under the following light and temperature regimes: 9-h photoperiod of 400 μmol photons m^-2^ s^-1^ under a day/night leaf temperature of 25°C/20°C (open symbols) or 14°C/8°C (light gray symbols), or 9-h photoperiod of 1000 μmol photons m^-2^ s^-1^ under a day/night leaf temperature of 25°C/20°C (dark gray symbols) or 14°C/8°C (black symbols). Mean values ± standard deviation, *n* = 3 to 4 leaves (one from each of three or four plants). The line is described by *y* = 1.15*x* - 36, *R*^2^ = 0.81, and *p* < 0.003.

Furthermore, a remarkable linear relationship existed between photosynthetic capacity and the product of phloem cell numbers and foliar vein density for *A. thaliana* and spinach (**Figure 3**). Phloem cell number, the number of all phloem cells of minor loading veins directly associated with sieve elements, was normalized for (multiplied by) foliar vein density since vein density was approximately twice as great in leaves of spinach versus *A. thaliana* (vein density did not vary within either of these species in response to growth conditions; see also Candela et al., 1999; Amiard et al., 2005; Adams et al., 2007). In both species, the number of phloem cells (those associated with sieve elements and the number of sieve elements themselves) and photosynthetic capacity both increased with decreased temperature and increased light intensity during growth (**Figure 3**). These findings suggest that higher photosynthetic capacities can be supported by higher levels of phloem loading (facilitated by a greater number of loading cells) and higher levels of sugar flux through greater numbers of sieve elements in minor loading veins of winter-active apoplastic loaders. While the intrinsic (light- and CO_2_-saturated) capacity of photosynthesis shows the closest correlation with various phloem vascular features (our unpublished data), there is also a close relationship between intrinsic photosynthetic capacity and *in situ* photosynthesis rates during the actual growth conditions. Our comparisons between intrinsic photosynthetic capacities and *in situ* rates of photosynthetic electron transport estimated from chlorophyll fluorescence parameters (not shown) indicate that (i) leaves grown at high versus low light levels exhibit similarly greater rates of both intrinsic photosynthetic capacity and photosynthetic electron transport under growth conditions and (ii) leaves of winter annuals grown at cool versus warm temperatures show an association between greater intrinsic photosynthetic capacities (measured at 25°C) and maintenance of similar high actual rates of photosynthetic electron transport under growth conditions, while leaves of cold-intolerant summer annuals show a failure to increase intrinsic photosynthetic capacities (measured at 25°C) associated with much lower actual rates of photosynthetic electron transport when grown under cool versus warm growth temperatures.

## VEIN DENSITY, AND POSSIBLY NUMBERS OF PHLOEM CELLS IN MINOR VEINS, ARE RELATED TO PHOTOSYNTHETIC CAPACITY IN SEVERAL SUMMER-ACTIVE APOPLASTIC LOADERS

In contrast to the winter-active apoplastic loaders *A. thaliana* and spinach that exhibited no variation in vein density in leaves that had developed under different growth conditions, the greater photosynthetic capacities of HL-acclimated leaves in the summer-active apoplastic loader tomato were associated with vein densities that were significantly higher compared to those of leaves that had developed in LL (Adams et al., 2007). Furthermore, a comparison of two apoplastically loading summer annuals grown under common conditions of HL revealed that sunflower (*Helianthus annuus* L. Soraya) possessed a greater number of companion cells and sieve elements in the minor veins (**Figure 2**), as well as a significantly higher vein density, compared to tobacco (*Nicotiana tabacum* L.; 9.0 ± 1.7 versus 4.2 ± 0.6 mm vein length per mm^2^ leaf area, mean ± standard deviation (SD), *n* = 4, *p* < 0.001, Student’s *t*-test). These foliar vein features may provide for a greater flux of photosynthate out of the leaves in support of photosynthetic capacities that were significantly higher in sunflower leaves compared to tobacco leaves (**Figure 2**).

## INTERMEDIARY (COMPANION) CELL SIZE AND VEIN DENSITY ARE RELATED TO PHOTOSYNTHETIC CAPACITY IN SEVERAL SYMPLASTIC LOADERS

**FIGURE 4:**
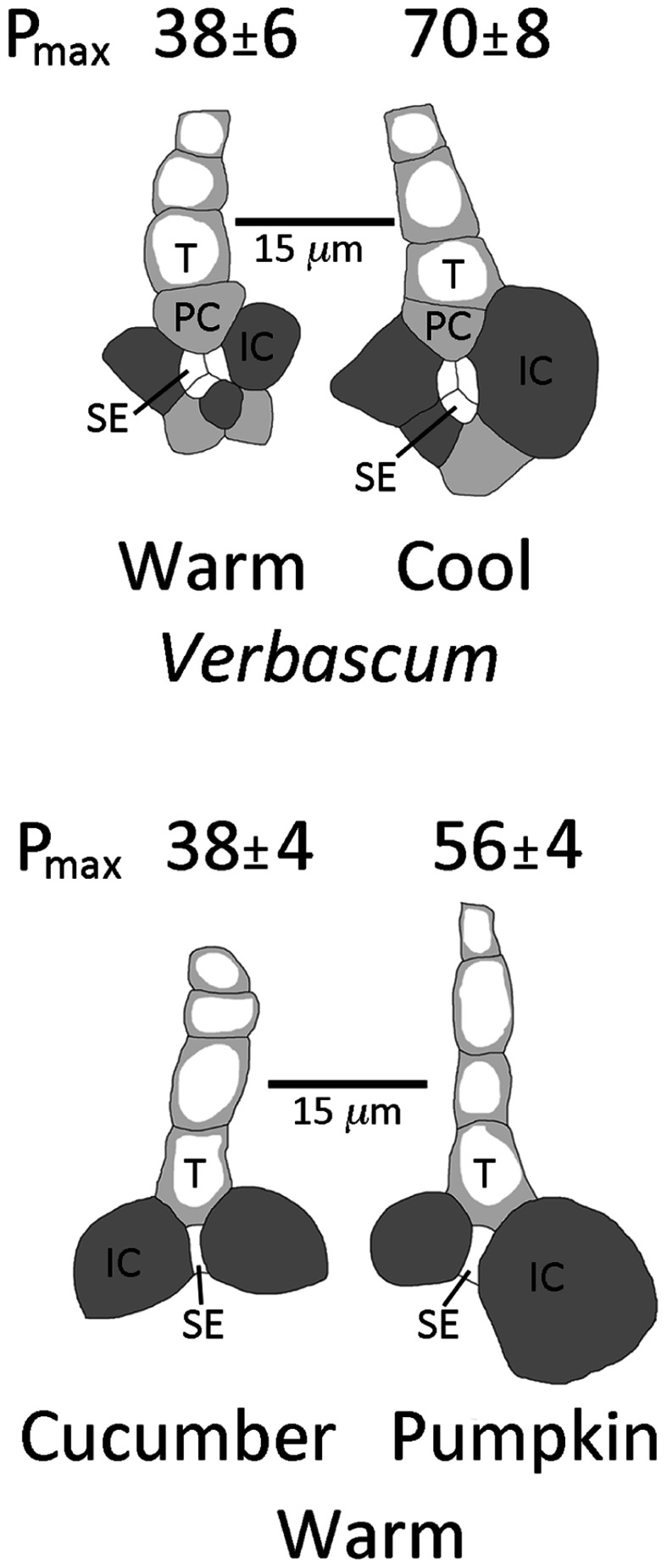
**Sketches (from light microscopic images) of representative minor loading veins from fully expanded leaves of *Verbascum phoeniceum* grown under a 9-h photoperiod of 400 μmol photons m ^–2^ s^–1^ at an average day/night leaf temperature of 25° C/20° C (warm) or 14° C/8° C (cool) and of minor loading veins from fully expanded leaves of cucumber and pumpkin grown under a 14-h photoperiod of 1000°mol photons m^–2^ s^–1^ at an average day/night leaf temperature of 25° C/20° C.** Filled dark gray cells are intermediary (companion) cells (IC), filled light gray cells are phloem parenchyma cells (PC), small white cells are sieve elements (SE), and cells with light gray adjacent to the cell walls are tracheids (T). Photosynthetic capacities (light- and CO_2_-saturated rates of oxygen evolution, *P*_max_ in μmol O_2_ m^-2^ s^-1^) ± standard deviation obtained from one leaf from each of four plants (*n* = 4) per growth condition provided above each sketch. Mean photosynthetic capacities between *V. phoeniceum* leaves grown at the two different temperatures, and between cucumber and pumpkin grown under the common conditions, were significantly different at *p* < 0.001 (Student’s *t*-test).

Under moderate growth light (400 μmol photons m^-2^ s^-1^), the intermediary cells of the biennial symplastic loader *V. phoeniceum* were larger in minor loading veins of leaves that had developed under cool temperatures (day/night leaf temperatures of 14°C/8°C) compared to leaves that had developed under warm temperatures (25°C/20°C; **Figure 4**). As mentioned above, such leaves also exhibited a greater vein density per leaf area when grown at lower versus warmer temperature (Dumlao et al., 2012). These differences in vein density and intermediary cell size are associated with photosynthetic capacities that are approximately twice as high in leaves grown at cool versus warm temperatures (**Figure 4**; Dumlao et al., 2012). There was furthermore an association between greater vein density and higher photosynthetic capacities for leaves of the symplastic loaders *V. phoeniceum* and pumpkin when both were grown under HL compared to LL at warm temperature (Amiard et al., 2005).

Similar general associations can also be seen between different symplastically loading species grown under common conditions. Two summer annual symplastic loaders, cucumber (*Cucumis sativus* L. cv. Straight Eight) and pumpkin (*Cucurbita pepo* L. cv. Autumn Gold), were grown under HL and warm temperatures. Under these conditions, pumpkin had a higher foliar vein density (9 ± 1 versus 6 ± 1 mm vein length per mm^2^ leaf area, mean ± SD, *n* = 4, *p* < 0.01, Student’s *t*-test), larger intermediary cells associated with the sieve elements of minor loading veins (**Figure 4**), and a significantly higher photosynthetic capacity (**Figure 4**) compared to cucumber. For all symplastic loaders examined here, higher photosynthetic capacities were thus correlated with (and presumably supported by) larger intermediary cells (presumably with more enzymes for synthesizing raffinose sugars) and higher foliar vein densities (to support a greater flux of sugars through, and out of, the leaf).

## THE INFRASTRUCTURE OF THE DISTRIBUTION SYSTEM MAY SET THE LIMIT ON SUGAR FLUX AND, THEREBY, SUGAR PRODUCTION CAPACITY

**FIGURE 5:**
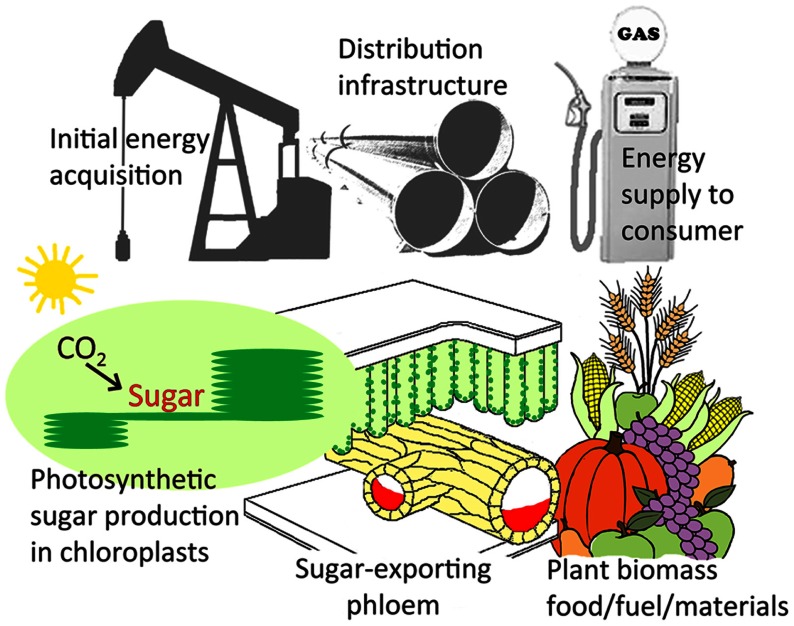
**Analogy between energy acquisition, distribution, and consumption for petroleum versus the sugar produced in photosynthesis.** Scheme illustrating the role of the infrastructure of the sugar-exporting phloem (illustrated in orange as the lower portion of the leaf veins) in distributing sugar from the chloroplasts in the mesophyll cells of the leaf (site of photosynthetic sugar synthesis) to the sites of sugar utilization for the production of agronomically important plant materials (food, fuel, and other products). The role of sugar-exporting phloem is analogous to petroleum distribution infrastructure, where limited distribution would be expected to put an upper limit on the investment into the acquisition of petroleum. For details regarding the cellular arrangement of the phloem in foliar minor loading veins, see **Figures 2** and **4** for representative examples from leaves with different phloem loading mechanisms.

The findings presented here suggest that active sugar loading, and the physical route for sugar export from sugar-producing leaves, constitutes a potential limitation to leaf photosynthesis – and that a greater capacity for phloem loading and for sugar transport in foliar sieve elements allows for a greater leaf photosynthetic capacity. In the economic analog presented in **Figure 5**, increased investment in industrial production would only be sustainable (profitable) if sufficient infrastructure were available for distribution of more products to the consumer. Likewise, increased investment by the plant in expensive-to-maintain photosynthetic machinery should only be expected if sufficient sugar export infrastructure is available to distribute the resulting increased sugar production to the plants’ sugar-consuming sinks.

Perhaps not surprisingly, different species with different evolutionary histories and different mechanisms for exporting soluble carbohydrates from their leaves exhibit different apparent approaches to providing a high sugar export infrastructure. In response to growth at lower temperature and/or higher light intensities, winter-active apoplastic loaders exhibited greater levels of cell wall invagination in transfer cells and/or greater total numbers of cells involved in active loading (both of which provide for a greater overall membrane area for trans-membrane proteins involved in active phloem loading), while a concomitant greater total number of sieve elements per minor loading vein provides the necessary cross-sectional area for a greater sugar flux capacity. While the winter-active apoplastic loaders exhibited no phenotypic plasticity with regard to foliar vein density, the summer-active apoplastic loader tomato developed a significantly greater vein density in HL compared to LL, and a comparison among two summer-active apoplastic loaders revealed a correlation between vein density and photosynthetic capacity. In symplastic loaders, the general architecture of the minor loading veins (numbers and arrangement of cells) exhibited little or no response to growth conditions, but cell size and foliar vein density did exhibit acclimatory differences. Growth of symplastic loaders at lower temperature (for a biennial species that persists through winter) and/or higher light intensities (for summer annuals as well as the biennial species) led to leaves with larger intermediary cells (with a greater volume in which to accumulate raffinose-synthesizing enzymes) and higher vein densities (to support a greater overall flux of sugars out of the leaves).

The two principal adjustments to accommodate greater sugar flux through the leaf’s sieve elements (greater number of sieve elements per minor loading vein versus greater total numbers of veins per unit leaf area) are also strategies that might work equally well in delivering more water through the xylem (greater number of tracheids per vein, cf. *A. thaliana* in **Figure 2**, versus greater density of foliar veins, e.g., Brodribb and Jordan, 2011) in support of the transpirational water loss that typically accompanies photosynthetic CO_2_ uptake through the stomata. It will be interesting to discover what adaptive and/or acclimatory (during leaf development) adjustments to the minor loading veins (if any) species from different major groups of plants (e.g., gymnosperms, monocots) and/or with other life histories (e.g., seasonally deciduous species, evergreen species) employ.

## Conflict of Interest Statement

The authors declare that the research was conducted in the absence of any commercial or financial relationships that could be construed as a potential conflict of interest.
